# Real-world treatment management in hereditary transthyretin amyloidosis – an experience report and proposal for therapy switch decision criteria

**DOI:** 10.1186/s42466-025-00428-6

**Published:** 2025-09-12

**Authors:** Duc Chu Dieu, Helena F. Pernice, Harisa Muratovic, Paul J. Wetzel, Gina Barzen, Nicolas W. Wieder, Stefanie M. Werhahn, Bettina Heidecker, Sebastian Spethmann, Katrin Hahn

**Affiliations:** 1 Amyloidosis Center Charité Berlin (ACCB), Charité University Medicine, Berlin, Germany; 2 Department of Neurology, Charité University Medicine, Berlin, Germany; 3 Department of Cardiology, Angiology and Intensive Care Medicine, Deutsches Herzzentrum der Charité, Charitéplatz 1, 10117 Berlin, Germany; 4 Charité – Universitätsmedizin Berlin, corporate member of Freie Universität Berlin and Humboldt-Universität zu Berlin, Charitéplatz 1, 10117 Berlin, Germany; 5 DZHK (German Centre for cardiovascular research), Partner site Berlin, Berlin, Germany; 6 Deutsches Herzzentrum der Charité, Department of Cardiology, Angiology and Intensive Care Medicine, Hindenburg Damm 30, 12203 Berlin, Germany; 7 Berlin Institute of Health at Charité – Universitätsmedizin Berlin, Charitéplatz 1, 10117 Berlin, Germany; 8 Deutsches Herzzentrum der Charité, Department of Cardiology, Angiology and Intensive Care Medicine, Augustenburger Platz 1, 10117 Berlin, Germany

**Keywords:** Hereditary transthyretin amyloidosis, Patisiran, Vutrisiran, Tafamidis, Inotersen, SiRNA, Tetramer stabilizer, ASO, Gene silencer

## Abstract

**Background:**

Hereditary transthyretin amyloidosis is a rapidly progressive and lethal disease. Thanks to the increasing number of disease-modifying treatments, prognosis has improved significantly. However, new challenges regarding treatment response and when to change treatment remain unanswered. The objective of this study was to evaluate rationales for treatment switches from the past and to formulate learnings for future management.

**Methods:**

In this retrospective single center study, we analyzed real-world data of 13 patients with hereditary transthyretin amyloidosis undergoing single or multiple treatment switches before January 2024. Data involved demographic characteristics as well as reasons for treatment switches in a descriptive and exploratory manner. Available amyloid specific therapies during the study period included tafamidis 20 mg, tafamidis 61 mg, patisiran, inotersen and vutrisiran.

**Results:**

Switches from tafamidis 20 mg were most frequently due to disease progression (83.3%). Patisiran transitions predominantly occurred following vutrisiran’s approval, driven by preference for subcutaneous administration and extended dosing intervals (65.0%). Two cases of switches from inotersen were both associated with severe adverse effects.

**Conclusions:**

In this study, reasons for treatment switches were manifold, encompassing disease progression, the occurrence of adverse events, patient preferences and/or the availability of newly approved drugs. Hence, multidimensional consideration of these reasons remains pivotal in guiding the subsequent choice of medication in particular and managing hereditary transthyretin amyloidosis in general.

## Background

Hereditary transthyretin amyloidosis (ATTRv-amyloidosis) is a rare autosomal dominant genetic disease characterized by multiple point mutations in the transthyretin (TTR) gene [1]. To date, over 150 mutations have been reported [2] – the most common being *p.Val50Met* mutation [3]. These mutations induce thermodynamic instability to TTR tetramers, causing dissociation into conformationally-altered monomers that oligomerize and misfold into amyloid fibrils [4, 5]. Subsequently, these fibrils deposit interstitially in various organ systems i.e. peripheral nerves and myocardium [6], driving progressive organ dysfunction [7, 8]. The resulting clinical phenotype most frequently presents as a rapidly progressive bilateral sensorimotor [9] and autonomic polyneuropathy [10] (neurological phenotype) and/or cardiomyopathy [6] (cardiac phenotype), with a combination being the most prevalent (mixed phenotype) [2]. Depending on the patient’s walking capacity, polyneuropathy severity in Familial Amyloid Polyneuropathy (FAP) can be staged as follows: symptomatic but fully ambulatory (stage 1), requiring walking aids (stage 2) and wheelchair dependence (stage 3) [11, 12]. Additionally, gastrointestinal [13], renal [14], ocular [15] and central nervous system [16, 17] involvements may also develop. Given the severe and heterogenous nature of the disease course, amyloid-specific treatment strategies are pivotal to stabilize progression. As of June 2025, a multitude of treatment strategies are available in the treatment of ATTRv-amyloidosis, ranging from: FDA/EMA-approved drugs [18–24], to off-label use of repurposed drugs [25] as well as novel agents currently under clinical development [26, 27]. While drug availability differs between regions and countries, this study focusses on FDA-/EMA-approved drugs targeting gene silencing [small interfering RNA (siRNA) and antisense oligonucleotide (ASO)] and tetramer stabilization (status: 01/2024).

By selectively targeting the complementary 3’ end of the mRNA of the *TTR* gene, siRNA induce posttranscriptional inhibition of gene expression and, consequently, a transient TTR knockdown. Patisiran (Onpattro™), an siRNA drug encapsulated in lipid nanoparticles for specific hepatic delivery and uptake [28, 29], received approval by FDA and EMA in 2018 for the treatment of FAP stages 1 and 2 [12]. Patisiran´s approval was rooted in APOLLO-A trial data (NCT01960348), where the verum group exhibited significant improvements in the modified Neurological Impairment Score (mNIS + 7) [30] and the Norfolk Quality of Life-Diabetic Neuropathy (Norfolk QoL-DN) [31] as well as TTR levels reduction of ∼ 80% as compared with placebo [28]. This precedent was mirrored in 2022 when vutrisiran, a second-generation siRNA drug with enhanced stabilization chemistry and N-acetylglucosamine conjugation, gained FDA-/EMA-approval [32]. In the HELIOS-A trial (NCT035759379), the group under vutrisiran met its primary endpoint, the mNIS + 7 at month 9 vs. placebo [19]. Vutrisiran also received approval for the ATTR-cardiomyopathy (ATTR-CM) in June 2025, based on the HELIOS-B trial (NCT04153149) [33].

Gene silencing may also be achieved via ASO, mediating RNA interference through RNase H-dependent mRNA cleavage [12]. Inotersen (Tegsedi™) received regulatory approval in 2018 for FAP stages 1 and 2 [12] based on its demonstrated efficacy in stabilizing the mNIS + 7 and Norfolk QoL-DN and reducing the TTR levels by ∼ 70% in the verum group of the NEURO-TTR trial (NCT01737398) [20]. Importantly, a second generation ASO agent, eplontersen (Wainzua™, Wainua™) (NCT01737398, NEURO-TTRansform), was approved in the United States in 2023 and in Germany in 2025, further increasing therapeutic options [22, 34].

Mimicking thyroxine properties to stabilize TTR tetramers when binding to one of its interdimeric binding pockets [35], transthyretin stabilizers kinetically inhibit TTR tetramer dissociation [36] as the rate-limiting step of amyloidgenesis [29]. Tafamidis 20 mg (Vyndaquel™, Vyndamax™) became the first FDA- and EMA-approved drug in the treatment of FAP stage 1 in 2011 [12], showing stabilization of the Neurological Impairment Score for Lower Limbs (NIS-LL) and the Norfolk QoL-DN in patients with *p.Val50Met* mutation at month 18 as compared with placebo in the landmark Fx-005 trial (NCT00409175) [24]. In 2019 (FDA) and 2020 (EMA), its indication was extended to include cardiomyopathy (ATTR-CM) at a 61 mg dose based on the reductions in all-cause mortality and cardiovascular-related hospitalizations vs. placebo in the ATTR-ACT trial (NCT01994889) [18]. Acoramidis (Beyonttra™, Attruby™), a new tetramer stabilizing agent [23] and Vutrisiran, were approved for ATTR cardiomyopathy in 2025 [33].

For seven years following its approval, tafamidis remained the sole FDA/EMA-approved amyloid-specific drug. With the expansion of the disease-modifying therapeutic landscape in the treatment of ATTRv-amyloidosis, comprehension of drug effectiveness and safety is pivotal for the initial choice of medication and algorithms for treatment switching and sequencing. However, trial designs of these drugs differed strongly by inclusion criteria, endpoints and study period [18–20, 24, 28]. Head-to-head comparisons of disease-modifying treatment strategies remain notably lacking. Furthermore, despite differences in approval for disease severity, no clear guidelines or recommendations for treatment initiation or switches exist. Hence, the aim of this study is to reflect real-world experience of patients undergoing treatment switching in furtherance of the evidence base regarding the efficacy, tolerability and practical application of these drugs in ATTRv-amyloidosis therapy.

## Methods

### Study design

This retrospective study was conducted at the Amyloidosis Center Charité Berlin (ACCB). The Medical Ethics Committee had approved this study as part of the Amyloidosis Register of the ACCB (EA1/014/020). All research was performed in accordance with the 1964 Declaration of Helsinki. Data were extracted from electronical hospital files of eligible patients with ATTRv-amyloidosis.

### Study population

Mandatory inclusion criteria were a proof of pathogenic mutation in the *TTR* gene, age > 18 years and informed consent for research purposes. We screened electronical hospital files from 01/2013 to 01/2024 retrospectively for eligible patients (*n* = 65). The analysis excluded therapy-naïve patients (*n* = 39). Consequently, the ATTRv-amyloidosis cohort comprised 13 patients who underwent therapy switching and a non-switch cohort (*n* = 13).

### Treatment decisions and Documentation

Treatment decisions were made by an interdisciplinary board including treating cardiologists, neurologists, gastroenterologists, nephrologists, ophthalmologists, and nuclear medicine specialists. Documentation was performed using the TBase platform [37].

### Outcome measures and statistical analysis

This study was conducted as a retrospective, observatory, exploratory study. Patient demographics and disease characteristics of the ATTRv-amyloidosis cohort were evaluated via descriptive statistics. Categorial variables were described as frequencies, metric variables via median and interquartile range (IQR).

Treatment switching was evaluated by analyzing specific reasons for discontinuation/switching and treatment timelines. Subjective neurological deterioration was assessed through patient´s self-reported perception of change since their last visit and standardized questionnaires [31, 38], whereas objective neurological change was measured using the Neurological Impairment Score (NIS) [30]. Quantitative analysis identified switch frequencies at the case level, with results clustered into clinically relevant categories and presented descriptively. Qualitative assessment further examined individual patient journeys, contextualizing statistical patterns with motivating factors and timeline.

Following the identification of treatment switches under tafamidis 20 mg due to neurological and/or cardiological progression the change in the NIS [points], Composite Autonomic Symptom Score-31 (COMPASS-31) [points] [39], N-terminal pro-B-type natriuretic peptide (NT-proBNP) [ng/L] and interventricular septum thickness enddiastolic (IVSd) [mm] were documented. The time frame of change [months] captures the span from the data point nearest to the switch from tafamidis 20 mg to the earliest point approaching treatment initiation under tafamidis 20 mg. For each parameter the median (IQR) for all available data points were described.

As comparison, we analyzed a non-switch cohort with a similar age distribution. In these patients, median changes (IQR) in the NIS [points], COMPASS-31 [points], NT-proBNP [ng/L] and IVSd [mm] were evaluated at month 12 ± 2 months since treatment initiation.

## Results

### Patient demographics and disease characteristics

**Fig. 1:**
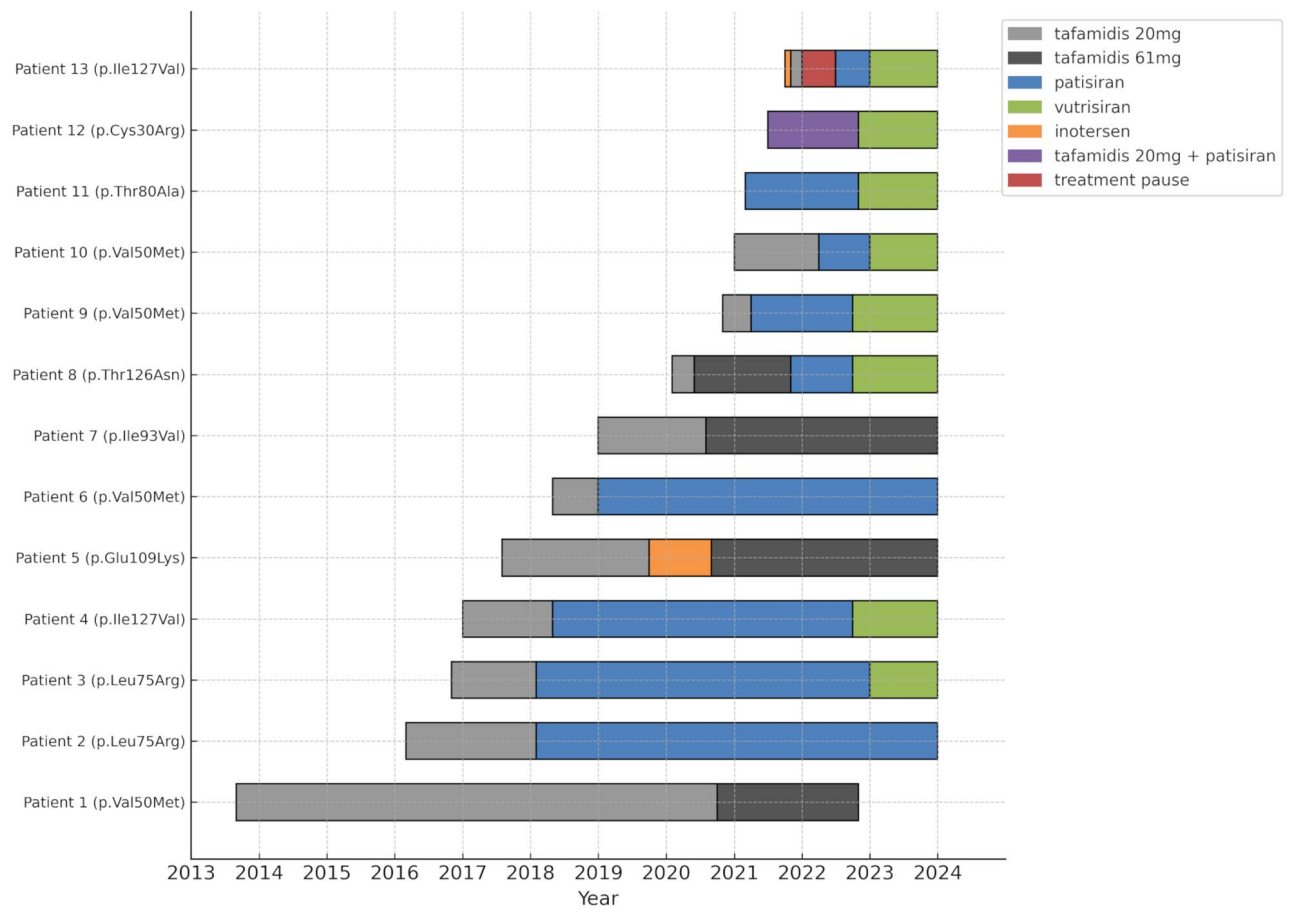
Treatment course in the ATTRv-amyloidosis cohort. Color codes depict disease-modifying agent and/or therapeutic approach. Grey = tafamidis 20 mg, dark grey = tafamidis 61 mg, blue = patisiran, green = vutrisiran, orange = inotersen, purple = tafamidis 20 mg + patisiran, red = treatment pause

The study cohort comprised 13 patients (100.0%) with hereditary transthyretin amyloidosis (Table 1). Of these, nine patients (69.2%) were male, and four patients (30.8%) carried the *p.Val50Met* mutation. The specific genotype spectrum is included in Fig. 1. All patients were symptomatic at initial presentation. The majority presented with neurological (61.5%) or mixed phenotypes (31.5%). No cases of isolated cardiac involvement were observed in this cohort. Patients began treatment at median age of 66.0 years (IQR 55.0–72.0), with a median interval of 24.0 months (IQR 12.0–60.0 months) between symptom onset and first treatment. Current treatment had been initiated at a median age of 70.0 years (IQR 56.0–76.0 years), following a median treatment duration of 24.0 months (IQR 12.0–36.0 months) of prior treatment with other agents.

**Table 1:** Patient demographics and disease characteristics

	ATTRv- amyloidosis cohort	*N* (%)
Number of patients, *n* (% of the entire cohort)	13 (100.0)	
Male, *n* (%)	9 (69.2)	
p.Val50Met, *n* (%)	4 (30.8)	
Age at disease onset [years], median (IQR)	61.0 (50.0–67.0)	13 (100.0)
Age at diagnosis [years], median (IQR)	66.0 (54.0–72.0)	
Symptomatic at initial presentation, *n* (%)	13 (100.0)	
Phenotype		13 (100.0)
Pure neurological, *n* (%)	8 (61.5)	
Pure cardiac, *n* (%)	0 (0.0)	
Mixed, *n* (%)	5 (38.5)	
Age at initial treatment initiation [years], median (IQR)	66.0 (55.0–72.0)	13 (100.0)
Time between disease onset and treatment initiation (any treatment) [months], median (IQR)	24.0 (12.0–60.0)	13 (100.0)
Initial treatment		13 (100.0)
tafamidis 20 mg, *n* (%)	10 (76.9)	
tafamidis 61 mg, *n* (%)	0 (0.0)	
inotersen, *n* (%)	1 (7.7)	
patisiran, *n* (%)	1 (7.7)	
vutrisiran, *n* (%)	0 (0.0)	
tafamidis 20 mg + patisiran	1 (7.7)	
Age at last treatment initiation [years], median (IQR)	70.0 (56.0–76.0)	13 (100.0)
Time of treatment before last treatment [months], median (IQR)	24.0 (12.0–36.0)	13 (100.0)
Treatment at end of observation period (2024)		12 (100.0)
tafamidis 20 mg, *n* (%)	0 (0.0)	
tafamidis 61 mg, *n* (%)	2 (16.7)	
inotersen, *n* (%)	0 (0.0)	
patisiran, *n* (%)	2 (16.7)	
vutrisiran, *n* (%)	8 (66.7)	

### Treatment switching

A total of 13 patients with hereditary transthyretin amyloidosis underwent singular or multiple therapy changes over the course of treatment. A total of 23 treatment switches were documented during the study period. The detailed treatment course is provided in Fig. 1 and the primary reasons for discontinuation or transition between therapies are summarized in Table 2.

No treatment discontinuations of vutrisiran were observed in our cohort (Table 2).

**Table 2:** Reasons for treatment switching in our cohort. 13 patients were switched from amyloidosis-specific treatment. Multiple reasons for drug switches May apply for one drug

	tafamidis 20 mg	tafamidis 61 mg	inotersen	patisiran
number of therapy switches, *n*	12	1	2	8
disease progression, *n* (%)	10 (83.3)	1 (100.0)	0 (0.0)	1 (12.5)
neurological progression, *n* (%)	8 (80.0)	0 (0.0)	0 (0.0)	1 (100.0)
cardiological progression, *n* (%)	5 (50.0)	1 (100.0)	0 (0.0)	0 (0.0)
adverse effects, *n* (%)	1 (8.3)	0 (0.0)	2 (100.0)	2 (25.0)
novel therapy and patient preferences, *n* (%)	4 (33.3)	0 (0.0)		6 (75.0)
preoperative adjustments, *n* (%)	0 (0.0)	0 (0.0)	1 (50.0)	0 (0.0)

Tafamidis 20 mg was the most common initial therapy (76.9%, Patient 1–10), while inotersen (Patient 13), patisiran (Patient 11), or a combination of tafamidis 20 mg with patisiran (Patient 12) were each administered to one patient (7.7% of all patients). Importantly, 60% of tafamidis 20 mg initiations occurred at the time it was the only FDA/EMA approved treatment.

Disease progression was the primary cause for switching from tafamidis 20 mg for 83.3% of patients (*n* = 10). Progression of neuropathy (median increase in NIS = 10 points over a median time of 16 months, see Table 3) was the primary cause in 3 patients: Patient 2 transitioned from tafamidis 20 mg to patisiran due to progressive polyneuropathy, significant weight loss, and gastrointestinal dysautonomia. Similarly, Patient 4 was switched to patisiran after developing progressive neuropathy and gait instability under tafamidis with an increase of the NIS by + 12.5 points, later transitioning to vutrisiran due to both continued neuropathic progression (+ 64 points in the NIS over the course of 46 months) and infusion-related side-effects. Patient 10 showed a + 34-point increase in the NIS and neurophysiological signs of an axonal-demyelinating neuropathy, leading to treatment changes from tafamidis to patisiran and finally to vutrisiran due to infusion-related side effects (cold sensation in lower limbs). Overall neurological deterioration observed in 80.0% of the ten cases (Patient 1, 2, 3, 4, 5, 6, 9, 10).

**Table 3:** Polyneuropathy and cardiomyopathy change as measured by NIS, COMPASS-31, plasma NT-proBNP and IVSd in patients that have been switched from Tafamidis 20 mg due to disease progression (*n* = 10). The time frame is defined retrospectively. M.V.=missing value. In patients with worsening NIS scores, sensory impairment was the most frequently affected domain, typically starting distally in the toes and progressing further over time. Reflexes were the second most impaired domain at baseline. Muscle weakness was generally absent at treatment initiation but appeared later in the disease course. These NIS findings should be regarded as qualitative patterns rather than results of formal statistical analysis

Patient	Polyneuropathy change				Cardiomyopathy change			
	NIS [points]	Time frame [months]	COMPASS-31 [points]	Time frame [months]	NT-proBNP [ng/L]	Time frame [months]	IVSd [mm]	Time frame [months]
	Switch primarily due to progressing neuropathy							
2	+ 3	14	M.V.	M.V.	M.V.	M.V.	± 0	14
4	+ 12.5	16	M.V.	M.V.	M.V.	M.V.	+ 1	13
10	+ 34	16	+ 0.89	M.V.	-37	16	-3	16
	Switch primarily due to progressing cardiomyopathy							
1	+ 52	84	+ 17.39	16	+ 1652	36	+ 8	60
5	± 0	27	+ 3.7	27	+ 1009	12	+ 2	27
6	+ 10	9	M.V.	M.V.	+ 1562	9	+ 4	9
	Other primary reasons							
3	+ 7	10	M.V.	M.V.	M.V.	M.V.	± 0	14
7	± 0	16	+ 0.33	19	+ 157	7	+ 0	19
9	+ 10	6	M.V.	M.V.	+ 36	6	-1	6
**Median (Range)**	**10 (0–52)**	**16 (6–84)**	**2.30 (0.33–17.39)**	**19 (16–27)**	**583 (-37-1652)**	**9 (6–16)**	**0 (-3-8)**	**14 (6–60)**

Additional or isolated cardiac progression was observed in 50.0% (Patient 1, 5, 6, 7, 8; median increase in NTproBNP = 583ng/l over a median time of 9 months, see Table 3). Patient 1, who received liver transplantation and tafamidis 20 mg, developed a significant pericardial effusion and progressive cardiomyopathy with an + 1652 ng/L increase in the plasma NT-proBNP over the course of 3 years and in IVSd changing from 19 to 27 mm over the course of 5 years before dose escalation to tafamidis 61 mg. Also, the NIS score increased by + 52 points over the full period of 7 years under tafamidis 20 mg. Ultimately, Patient 1 deceased to complications of prolonged septic shock with subsequent multi-organ failure in the setting of post-liver transplant immunosuppression. Patient 5 was switched from tafamidis 20 mg to inotersen after progressive cardiac decompensation and gastrointestinal manifestation, evidenced by an increase in the plasma NT-proBNP by + 1009 ng/l during the last year before switching and a + 3.7-point increase in the COMPASS-31 score, reaching a score of 40.3 points during tafamidis 20 mg therapy. Switching Patient 6 from tafamidis 20 mg to patisiran was prompted by a deterioration of plasma NT-proBNP (+ 1562ng/l) and IVSd by + 4 mm to 22 mm and a neurological deterioration as measured by a + 10-point increase in the NIS within 9 months under tafamidis 20 mg. Patient 8 was switched a first from tafamidis 20 mg to 61 mg due to availability of the higher dose, then patisiran due to escalating symptoms of heart failure and polyneuropathy (walking distance reduced to < 150 m, + 18-point increase in the COMPASS-31 score to 45.3 points at month 17 after tafamidis 60 mg initiation) and finally vutrisiran due to poor venous access.

Switches from inotersen (*n* = 2) were solely associated with adverse effects. Beforementioned Patient 5 developed thrombocytopenia, glomerulonephritis and hemorrhagic cystitis, requiring cessation of therapy and temporary re-initiation of tafamidis 61 mg, before then receiving a heart transplantation. Patient 12 experienced transient delirium and word-finding difficulties under the therapy with inotersen.

Apart from Patients 4 and 10 experiencing infusion-related reactions (nausea, dizziness, abdominal pain and cold sensation in the lower legs after infusion, respectively; representing 25.0% of patients), patisiran transitions to vutrisiran predominantly reflected the patients’ preference of subcutaneous administration and extended dosing intervals (65.0%, *n* = 6) (Patient 3, 8, 9, 11, 12, 13). Patient 3, for instance, opted for vutrisiran after long-term patisiran therapy, citing the psychological strain of frequent intravenous infusions.

At the end of the observation period, there was a predominance of vutrisiran (66.7%, *n* = 8) (Patient 3, 4, 8, 9, 10, 11, 12, 13). Two patients were treated with patisiran (Patient 2, 6), and two with tafamidis 61 mg (Patient 5, 7).

The non-switch (*n* = 13; three on tafamidis 20 mg, seven on tafamidis 61 mg, and three on vutrisiran) and ATTRv-amyloidosis cohorts were comparable in terms of age [non-switch cohort: 67.0 years (IQR 57.5–75.0)] but differed in sex distribution (non-switch cohort: 38.5% male). Over a follow-up of 12 ± 2 months, all assessed parameters remained remarkably stable: NIS 0.0 points (IQR − 2.0–0.0; *n* = 8), COMPASS-31 1.03 points (IQR − 11.03–6.44; *n* = 8), NT-proBNP 0.5 ng/L (-73.0-147.8; *n* = 10) and IVSd 0.0 mm (IQR − 0.8-2.0; *n* = 8).

### Proposal for treatment switch considerations

**Fig. 2:**
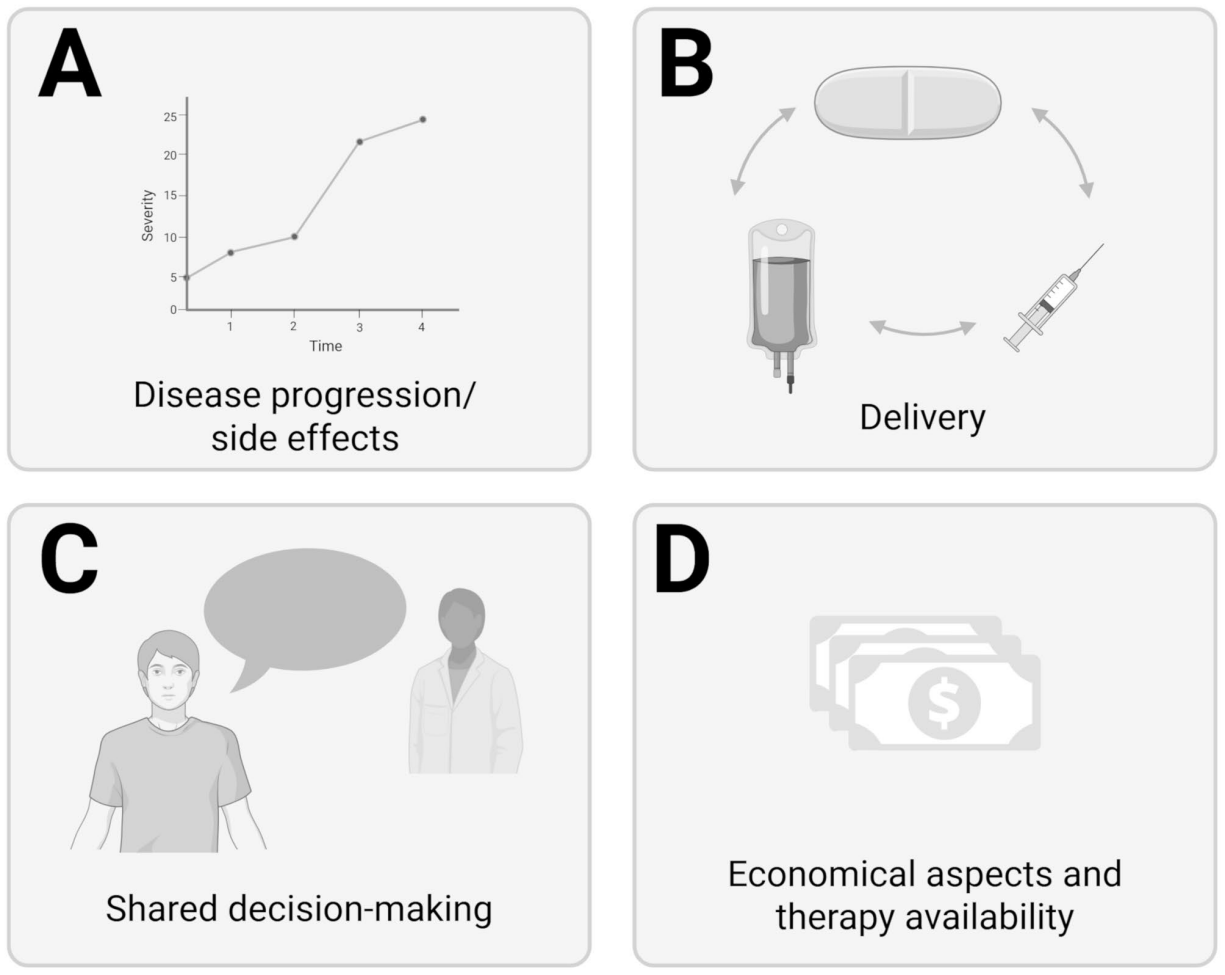
Proposal for key considerations for treatment switch based on experience in our cohort

Based on the beforehand mentioned data, we developed a proposal for treatment switch criteria (Fig. 2). First, side effects and clinically relevant change of symptoms should be examined and documented (A). Following the approval criteria for available drugs, patients should be evaluated for progression of cardiomyopathy and neuropathy: If mixed phenotypes exist, agents with approval for both cardiomyopathy and neuropathy should be preferred. Importantly, no specific score is required to justify treatment switches for insurances, and we therefore deliberately refrained from defining a single mandatory score or threshold for quantifying disease progression.

Second, clinical implications of delivery should be considered (B). For this, liver enzymes, platelet counts, and kidney function should be evaluated. Factors such as poor venous state or swallowing difficulties may impact choice of drug delivery.

Third, the patient preference should play a relevant role (following the principle of shared decision making) (C). This may include patient participation (ability to come to centers for treatments and to organize drug supply).

Last, newly available substances should be transparently discussed with the patient (D). Importantly, all available drugs are connected to considerable costs, not all therapeutic options may be available in all countries and economic circumstances may impact drug availability.

After considerations of above-mentioned criteria within the group of available treatment options, individualized therapeutic decisions should therefore be a result of an interdisciplinary discussion and justification facing the respective cost bearer.

Finally, these considerations require regular visits regarding to monitor therapy response and life circumstances.

## Discussion

In this retrospective single-center study, we analyzed reasons for treatment switches in ATTRv-amyloidosis patients. The varied courses and reasons illustrate the complexity of therapeutic decision-making.

While the initial choice of medication is primarily based on disease severity (i.e., FAP stage), this is the first report of experience regarding multi-therapy switches with different mechanisms of actions to our knowledge.

In our cohort, the most important reason for therapy switches was disease progression. In accordance to these finding, the German/Austrian position recommends an early switch to an alternative treatment modality when disease progression occurs [12]. For instance, the authors recommend that clinicians should not wait until near-FAP stage 2 progression before considering a switch – instead, close monitoring of symptoms, disease progression, and treatment response is essential to determine the optimal timing for therapy modification. Likewise to our experience, the choice of drug agents in transitions from tafamidis should be guided by patient-specific risk factors, administration preferences and tolerability profiles [12]. Tafamidis 20 mg was predominantly discontinued due to disease progression in our cohort, which reflects previous reports as measured by the increase of NIS scores over time [40–43]. In regard to efficacy of siRNA therapy, our results show that siRNA therapy may improve disease course in patients progressing under tetramer stabilizers with congruent findings in the literature [44]. Specifically, preliminary real-world data from Labeyrie et al. with a focus on the therapeutic outcome under patisiran after prior use of tafamidis was able to show similar reasons for treatment discontinuations under the tafamidis [44]. While progression of neuropathy was the reason for switching to patisiran in all 24 patient cases, cardiac progression accounted for 12.5% of treatment switches from tetramer stabilizers to siRNA. Importantly, Labeyrie et al. concluded that disease progression as measured by the NIS and PND scores was slower under patisiran than it was under tafamidis [44]. Our experiences underscore that despite patisiran’s proven efficacy for the treatment of ATTRv-amyloidosis, its administration protocol may impact long-term tolerability and patient adherence. Hence, switches to vutrisiran as an alternative siRNA agent were frequent in our cohort, allowing for subcutaneous administration every three months. The fact that in our control cohort without therapy switches, all assessed outcome markers (laboratory and clinical) remained stable, independent of the agent’s mechanism, underlines that individual assessment of therapy effect is essential for detection of potential non-responders and indication for therapy switch.

Other reasons for a treatment switch in our cohort were adverse effects and infusion-related side effects: For inotersen, adverse effects observed (glomerulonephritis and thrombocytopenia) align with safety findings from the NEURO-TTR trial, which reported three cases each of thrombocytopenia and glomerulonephritis [20]. Similarly, infusion-related reactions as seen in our cohort are a well-documented adverse effect of patisiran. As a standard preventive measure, premedication with paracetamol, prednisolone, H1- and H2-receptor blockers is routinely administered [12], prolonging the overall duration of frequent intravenous administration (every 3 weeks) and potentially introducing side effects attributable to the premedication.

Several therapeutic decisions for therapy switches were possible thanks to approval of new agents in Germany during the study period. This included dose escalation of tafamidis (from 20 mg to 61 mg) for patients demonstrating cardiac progression in our cohort at the time of approval of tafamidis 61 mg for the treatment of ATTR-CM. This approach is mechanistically supported by evidence that doses exceeding 60 mg achieve superior kinetic TTR stabilization [18]. Similarly, the availability of vutrisiran allowed switches from frequent intravenous to patient-preferred infrequent subcutaneous treatments of the same drug agent. However, current and future new approvals, also opening indications to different phenotypes, such as the recent approval of vutrisiran for cardiomyopathy in Germany [33] and the ongoing investigation of eplontersen for cardiomyopathy [45], impose new challenges not only on which treatment to use in which patient or when to switch to which agent, but also significant economic considerations in private and public scale (depending on insurance systems in place). Similarly, economic aspects may influence availability and guidelines on first-line therapies, limiting the drug and switch options:

For example, regional differences exist in the current use of vutrisiran vs. patisiran. The UK´s National Institute for Health and Care Excellence endorsed vutrisiran as first-line therapy over patisiran based on superior cost-effectiveness, while France´s Haute Autorité de Santé relegated vutrisiran to second-line status due to absence of demonstrated clinical superiority over patisiran [32].

Our study had important limitations. As a single center study, patient numbers were small, restricting generalizability of our real-world evidence. Therapeutic options available in the study period are rapidly changing and do not reflect global standards since they were based on approval in Germany. Moreover, due to the nature of a retrospective observatory study, patients were treated based on clinical decisions and indications without prior randomization or placebo groups. Differences in sex distribution between the switch and non-switch cohorts may have influenced disease course, and this should be considered when interpreting the findings.

## Conclusions

Ultimately, treatment switches in ATTRv-amyloidosis are prevalent and the underlying rationale is multifaceted, encompassing disease progression, adverse effects, patient preferences, and the availability of newer treatments with FDA/EMA approval. Our study found that neurological disease progression was the primary reason for therapy switches. While the availability of disease-modifying treatment options has expanded significantly, the absence of evidence-based guidelines for therapy switching and sequencing remains a major clinical challenge. There is an urgent need for prospective real-world, long-term and/or multi center studies comparing treatment strategies, development of validated biomarkers to guide therapeutic decisions as well as consensus guidelines incorporating real-world evidence to consolidate the evidence base of ATTRv-amyloidosis.

## Data Availability

The datasets of this study are available from the corresponding author on reasonable request.

## Abbreviations

ACCB: Amyloidosis Center Charité Berlin
ATTR-CM: Transthyretin amyloid cardiomyopathy
ATTRv-amyloidosis: Hereditary transthyretin amyloidosis
FAP: Familial Amyloid Polyneuropathy
IQR: Interquartile range
IVSd: Interventricular septum thickness
mNIS + 7: Modified Neuropathy Impairment Score + 7
NIS: Neuropathy Impairment Score
NIS-LL: Neuropathy Impairment Score for Lower Limbs
Norfolk QoL-DN: Norfolk Quality of Life-Diabetic Neuropathy
NT-proBNP: N-terminal pro-B-type natriuretic peptide, PND score: Polyneuropathy Disability score
TTR: Transthyretin
